# Social Connection and Online Engagement: Insights From Interviews With Users of a Mental Health Online Forum

**DOI:** 10.2196/11084

**Published:** 2019-03-26

**Authors:** Jennifer Smith-Merry, Gerard Goggin, Andrew Campbell, Kirsty McKenzie, Brad Ridout, Cherry Baylosis

**Affiliations:** 1 Centre for Disability Research and Policy Faculty of Health Sciences The University of Sydney Lidcombe Australia; 2 Department of Media and Communications Faculty of Arts and Social Science The University of Sydney Sydney Australia; 3 Cyberpsychology Research Group, Faculty of Health Sciences The University of Sydney Sydney Australia

**Keywords:** internet, mental health, social stigma, self-help groups, qualitative research, mental health recovery, mental disorders

## Abstract

**Background:**

Over the past 2 decades, online forums for mental health support have emerged as an important tool for improving mental health and well-being. There has been important research that analyzes the content of forum posts, studies on how and why individuals engage with forums, and how extensively forums are used. However, we still lack insights into key questions on how they are experienced from the perspective of their users, especially those in rural and remote settings.

**Objective:**

The aim of our study was to investigate the dynamics, benefits, and challenges of a generalized peer-to-peer mental health online forum from a user perspective; in particular, to better explore and understand user perspectives on connection, engagement, and support offered in such forums; information and advice they gained; and what issues they encountered. We studied experiences of the forums from the perspective of both people with lived experience of mental illness and people who care for people with mental illness.

**Methods:**

To understand the *experience* of forum users, we devised a qualitative study utilizing semistructured interviews with 17 participants (12 women and 5 men). Data were transcribed, and a thematic analysis was undertaken.

**Results:**

The study identified 3 key themes: participants experienced considerable *social and geographical isolation*, which the forums helped to address; participants sought out the forums to find a *social connection* that was lacking in their everyday lives; and participants used the forums to both find and provide *information and practical advice.*

**Conclusions:**

The study suggests that online peer support provides a critical, ongoing role in providing social connection for people with a lived experience of mental ill-health and their carers, especially for those living in rural and remote areas. Forums may offer a way for individuals to develop their own understanding of recovery through reflecting on the recovery experiences and peer support shown by others and individuals enacting peer support themselves. Key to the success of this online forum was the availability of appropriate moderation, professional support, and advice.

## Introduction

### Background

Web-based interventions for mental ill-health are increasingly becoming a part of policy frameworks for community mental health (eg, by the Australian and Scottish governments [1-3]), and large and well-used forums have been developed in many countries. Internet-based supports and services make sense for governments, given the number of people who incorporate the online into their social worlds and the considerable difficulties services and supports have accessing people face-to-face due to distance and other forms of social isolation. Previous studies have shown that although the rate of people in the general population accessing online mental health forums may be small, the proportion of people with diagnosed mental illness accessing forums is relatively high [4-6]. Yet, we lack essential information about how mental health forums are experienced by people with lived experience and the carers or what motivates their use, particularly for large generalized mental health forums that cater to different diagnoses. To fill this gap, this paper reports on interviews with people who are members of the SANE Forums, a well-used Australian online forum run by the nongovernment mental health organization SANE Australia. The research is part of a wider study exploring experiences of engagement with and use of the SANE Australia online forums with funding provided by the National Mental Health Commission. The aim of this paper is to address gaps in our knowledge about the experiences and motivations of forum use from the perspective of those who are regular users to provide evidence to shape their future development.

To provide a framework for the interviews, a literature review of existing studies was undertaken. The review included only studies that were concerned with online forums focusing on mental health. It did not focus on forums for nonmental health–related illnesses or discussions via social media. This review showed that studies focused primarily on a number of areas, including forums for specific diagnoses [7,8]; clinician-led or medical service–led sites, which are often used as an adjunct to other interventions [9-12]; and sites that focus on specific consumer groups including men and young people [13-15]. Analysis focused on 2 main areas: analyses of the content of forum posts and motivations and impact of forum use, but very little of this research focused on experiences from the forum user perspective. This existing literature is outlined here.

#### Content-Based Research

Research on the content of mental health–specific online forum postings has attempted to understand *illness* experiences or the construction of online chat rather than the user experience of accessing the forums. This includes, for example, the study of the experience of obsessive-compulsive disorder [7], cannabis addiction [16], dual diagnosis [17], and eating disorders [18]. Other content analysis has sought to determine whether forums help or hinder people’s recovery. Kavuluru et al [19] have developed a system to identify the helpfulness of comments on Reddit forums for mental health. Similarly, Cohan et al [13] drew on content from the Australian-based Reachout.com forums for young people to develop a matrix identifying harmful content so that it could be targeted and addressed more easily by forum moderators. Several studies have also attempted to characterize the types of posts that are made [20-22]. For example, a paper by Mazur and Mickel [23] coded content according to purpose and found that 86% of posts in a carer forum were about seeking advice or information on a specific topic and 20% were seeking support for the poster’s situation. Other content-based research utilizes forums as a methodological tool to understand naturally occurring chat for groups that may be difficult to engage with through traditional research. In addition, 2 studies, for example, analyzed men’s mental health, including a study which focused on depression [15] and another on eating disorders [8].

#### Impact of Forum Involvement

Existing studies on the “experience” of utilizing forums focus on positive or negative personal behaviors and emotions as a result of forum interactions. The impact of involvement in mental health–related online forums is shown to be largely positive in terms of symptom alleviation [18,24,25]. A small number of studies have warned of an increase in self-harm or mental distress due to forum use [26]. Interviews with people using a postnatal mental health support forum, for example, found that women could sometimes become more unwell because they were talking about the issues more [27]. These effects are viewed as particularly concerning in relation to unmoderated forums [28,29] and those that discuss eating disorders or suicide where techniques for harm are discussed [8]. One study found that negative forum experiences came from poor interactions with other forum members or disagreements with forum moderators [20]. Horgan et al [12] conducted a survey with 118 university students aged from 18 to 24 years with depression, who had been chosen to join a peer support forum. They found no benefit in symptom alleviation from involvement in the forum.

These studies largely focus on the impact of forum interactions on symptoms. However, studies that focus on symptom alleviation may miss the purpose of such forums, which are focused more on peer support for people moving toward recovery [30]. Recovery in this context does not mean to get better but to live a meaningful life with or without the symptoms of mental ill-health [31]. Peer support is an important component of recovery because it allows for modeling of recovery and provides hope for people who may be struggling with their mental ill-health [32]. Several papers reflecting on online forums used the structured recovery framework and determined that the forums did support participants’ recovery [20,33].

#### Motivations for Use

A small number of papers have examined the motivation of participants engaging in peer support via online forums or what they perceive they benefit from involvement. A survey reported by Seward et al [22] characterized the motivations of forum participants into 3 groups—those who sought online professional help, those who sought online anonymous help, and those who sought both online help and friendship. One paper provided a content analysis of posts to determine the utility of an online forum for participants by examining posts that reflected on the forum itself [20]. This study found that users believed that their involvement resulted in positive “personal change...social interactions and support.” A survey by Jones et al [34] of young people accessing the self-harm forum, *SharpTalk*, found that participants preferred to discuss self-harm anonymously online than with friends and family. An even smaller number of papers has involved qualitative interviews with forum participants. Moore and Ayers [27] drew on interviews with forum users seeking postnatal mental health support in the United Kingdom. They found that many forum users do not seek help in real life because of stigma but found the online forums helpful because they were anonymous, provided connections with others with similar experiences, and were nonjudgmental. This lack of interview-derived data is significant because data from surveys or content analysis of posts do not allow for in-depth exploration, contextualized in an individual’s life, of why people are motivated to use forums and what their experiences are.

What emerges from this examination of existing research is that there is a clear lack of in-depth, qualitative accounts of experiences of forum use in the context of an individual participant’s life. This paper addresses this gap by offering an understanding of the motivations for ongoing forum involvement among registered users of a large moderated forum for carers and consumers. As shown here, very few studies focus on the large-scale general forums, which are most accessible across a broad population base. Those that do focus on such forums do not focus on the experiences of individuals from their own perspective. Existing forum-based studies on other types of long-term conditions which do this are not fully applicable to the context of mental illness, which have been shown to differ both in the content of posts and the social context of the illness [35].

## Methods

### Context and Research Aims

The research presented here is part of a wider study exploring experiences of engagement with and use of the SANE Australia online forums.

To understand the *experience* of forum users, a qualitative design utilizing semistructured interviews was adopted to allow for a back-and-forth dialogue to foster and develop an understanding between participant and interviewer [36]. A topic guide was developed to guide the interviews with questions focusing on why participants used the forum, how they used it, what they liked and did not like about it, and how these issues could be addressed in forum design. The interview questions were designed to be as open as possible to provide practical information for the development of the forum. A copy of the topic guide is available from the lead author on request. All interviews were conducted by phone at a time convenient for participants. Each participant received Aus $30 gift card as compensation for their time in participating in the research. Phone interviews were chosen for practical reasons because participants were from across Australia and costs to interview in person would have been prohibitive. All participants were sent their interview transcripts and offered the opportunity to remove any details that they did not want to be included. Ethics approval was gained from the University of Sydney Human Research Ethics Committee (approval no. 2016/717). Interviews were conducted by KM, who is a psychologist and social science researcher and has worked as a postdoctoral qualitative researcher for several years. See Multimedia Appendix 1 for the semistructured interview schedule.

The forums used by participants in this study are operated by SANE Australia, are free to access, and are intended for Australian participants, although, in practice, are accessed by people worldwide. There are separate forums for carers and people with lived experience of mental ill-health, though this does not restrict who can publish on the forums. Within each forum, there are a number of subcategories of posts, with the *Lived Experience* forum including, among others: “our experience and stories,” “looking after our wellbeing,” “social spaces,” “something’s not right,” and “what’s new: services, research and technologies.” There are special forum sessions offered each week for discussion around topics that SANE identifies.

The forums operate under the 3 broad principles of respect, safety, and anonymity, and posting guidelines limit the content of posts. Participants are encouraged to “share helpful content, focused on wellbeing, recovery and help seeking behaviours” [37]. There are forum guidelines that require members to not post information about specific harmful behaviors and not provide details of medication. Any information provided must be from personal experience or trustworthy sources with links provided. Participants must use pseudonyms and must not share personal details about themselves. The forums do not offer crisis support but are constantly moderated by mental health professionals who monitor content and offer advice.

### Sampling

An initial survey, advertised to forum members by SANE, was conducted to understand who the SANE forum members were and how they used the forums (the survey results will be reported elsewhere). Consumers and carers were able to specify on the survey whether they were interested in participating in an interview. A total of 104 survey participants indicated their willingness to participate in an interview. A purposive sampling strategy was then used to identify a broad, but not statistically significant, range of consumer and carer participants, ensuring that those participants were included who were from both rural and metropolitan areas and a range of Australian states, genders, and ages. Although there were constrictions on the numbers of men sampled, given that all eligible male participants were included, no further female participants were included once the initial coding and theme development (see below) had been conducted because saturation had been reached.

**Table 1:** Participant characteristics.

Gender	Age (years)	Location (including state of Australia)	Forum
Female	26-30	Metropolitan South Australia	Lived experience
Female	31-35	Rural New South Wales	Carers
Female	31-35	Metropolitan Queensland	Lived experience
Female	41-45	Rural Victoria	Lived experience
Female	46-50	Rural Queensland	Both
Female	46-50	Rural Victoria	Lived experience
Female	46-60	Metropolitan New South Wales	Both
Female	51-55	Metropolitan Victoria	Both
Female	51-55	Rural Victoria	Lived experience
Female	51-55	Rural Queensland	Lived experience
Female	51-55	Rural Victoria	Carers
Female	56-60	Metropolitan Queensland	Lived experience
Male	26-30	Metropolitan New South Wales	Lived experience
Male	41-45	Rural New South Wales	Lived experience
Male	51-55	Australian Capital Territory	Lived experience
Male	51-55	Metropolitan Victoria	Lived experience
Male	61-65	Metropolitan Victoria	Carers

Participants were excluded if they were not based in Australia, did not provide valid contact details, or did not respond to researcher invitations to participate. Characteristics of the 17 participants are listed in Table 1. Only 12 men offered to participate, so the final number of male participants was lower (the remaining 7 were either not from Australia or did not provide contact details). Several people identified as both a carer and someone with a lived experience of mental ill-health and so used both forums. Only 3 of the participants who indicated they were interested in being interviewed and were aged 61 years and over were contactable. Of the 3, we were able to interview 1. Interviews concentrated on those who used the lived experience forum, but we also interviewed 3 respondents who used the carer’s forum and 3 people who indicated they used both forums.

### Analysis

Data were transcribed verbatim and entered into NVivo 10, where they were coded by hand.

Analysis of data followed a basic thematic approach to coding derived from that described by Braun and Clarke [38]. This involved the following 6 steps:

1. Step 1: Data familiarization—each interview transcript was initially read through several times to develop an initial sense of the themes that were emerging.
2. Step 2: Development of first set of codes—data were systematically coded in a line-by-line fashion according to the overarching research questions, described above with reference to the interview transcript.
3. Step 3: Theme development—codes were then sorted into draft thematic groups around key findings.
4. Step 4: Review of themes—a thematic map was developed and reviewed in relation to the initial full dataset. Transcripts were reread to check that the themes reflected the corpus as a whole.
5. Step 5: Definition of key themes—each theme was brought together as a set of data and explained to get a sense of its meaning as a whole.
6. Step 6: Reporting—the themes were developed into a narrative, which both explained the themes and provided evidence from the data in the form of quotations. The themes were presented in relation to existing research.

Throughout the data collection and analysis process, the researcher collecting data (KM) engaged in personal reflective practice by exploring her own emotional reactions to the experiences shared by participants. Given the nature of the research topic, participants often shared very difficult aspects of their lives, including suicidal ideation, hospitalization for mental ill-health, and the ongoing experience of social isolation and loneliness. It was felt important to acknowledge that hearing about and analyzing these experiences was not a neutral experience for this researcher. KM took care to make note of her emotional reactions to explore how these might impact the interactions during interview and subsequent analysis of data. Data analysis was primarily conducted by KM, but an independent cross-analysis of coding and theme development and reporting was conducted by JSM at steps 2, 4, and 6. This confirmed the initial coding. Both KM and JSM have extensive postdoctoral experience in qualitative research and teaching in universities. All authors checked the data and its analysis once an initial draft was complete. This allowed the expertise of the other authors (in cyber-psychology, media, and communication) to be used as a reflective tool. As a team, we worked through areas of analysis that were questioned from these alternative research standpoints.

## Results

### Overarching Themes Emerging From the Data

Analysis of the data revealed findings clustered around the following 3 key themes:

1. Participants experienced considerable *social and geographical isolation*, which the forums helped to address
2. Participants sought out the forums as a way to find a *social connection* that was lacking in their everyday lives
3. Participants sought out the forums to both find and provide *information and practical advice.*

### Experience of Mental Illness: Social Isolation and Geographical Isolation

#### Social Isolation

Participants described their lives in a variety of negative terms including loneliness, social isolation, stigma, exclusion, and workplace discrimination. This is noted by C08 who stated “Yeah, you know, you have the loneliness, you don’t know where to go, don’t have support...those stories just keep popping up. It’s scary how consistent it is.” Many participants felt unable to discuss mental illness in their everyday lives and communities because they felt that people were not interested, did not understand, or might hold prejudices and stigmatizing attitudes toward them. Several participants described feeling unable to leave the house due to their mental health symptoms or how these symptoms might appear to others.

They felt that people in their lives who had not experienced mental ill-health could not understand their experiences and, therefore, could not offer support:

...other than professionals, I haven’t had many people I can have conversations about mental illness with in my life, because unless they’ve been through it, they don’t reallythey can’t relate to it at all.

C11

You can’t just talk to anyone about mental health issues. They just don’t get it...They just don’t get it because their lives are so full.

C03

For others, this was related to stigma in their own communities or workplaces (C01, C05, and C15):

...after they find out I had a mental illness, they don’t want to be friends anymore.

C15

I get really upset about it because where I previously worked, they knew what happened to me while I was in hospital and they said, we don’t care, we’re not interested, we should have sacked you.

C01

The consequence of the isolation caused by a lack of understanding was profound, leading to loneliness and further mental distress. For these participants, the forums were a space where they could step out of the stigmatization and loneliness that they experience in their everyday lives into a community where mental health was accepted and understood. This is discussed further below.

Social isolation and inability to talk about mental illness appeared to be compounded for participants who lived in rural communities (C02, C05, C06, and C07). One participant spoke about the necessity to “hide” their illness and the lack of resources available to address mental ill-health in their own community:

When you’re in a country town you have to sort of hide it because people are not tolerant...I don’t have access to a lot of stuff and there’s nothing really out there to communicate with other people about your mental health.

C05

For this participant, the forums offered an alternative space for connection that helped to reduce the social isolation they experienced in their rural community:

I think it’s helped me because it’s a connection...I’m in a restricted country area and I’ve hurt my back so I don’t work in nursing. So I’m home all day. It’s just company to know that there’s other people out there.

C05

Rural participants faced additional barriers in accessing supports in their area. This was true for carers as well as those with a lived experience of mental illness (C06). In 1 instance, a participant mentioned that although services were available in her area, she knew all the psychiatrists because she used to work in mental health so did not feel comfortable accessing them (C04). She, therefore, had to travel to a capital city to access support. Another mentioned that the only face-to-face social support groups in his area were for people with specific needs and did not cater to chronic mental illness (C02). Another said that in their rural area, there was only 1 support group and timing was not appropriate for those with young children (C06). For these participants, the forums offered a support group that they could access when they wanted and for their own specific needs.

#### Social Connection

As the following quotations demonstrate, for participants who were socially isolated and unable to speak about mental illness or connect with anyone with similar experiences in their own communities, the forums offered an important opportunity for social connection:

...I can always get online and so that’s the beauty of it.

C05

...for me it’s a real connection. I’m not the only one with bipolar disorder.

C07

The forums also provided an opportunity to reframe experiences, receive support, and give back or help others who were struggling with the same issues.

Participants described accessing the forums in an attempt to ameliorate their social isolation. The forums presented an opportunity to discuss the reality of their situation when it was not possible or appropriate to discuss their experiences with others. They spoke of “struggling” (C12) to speak to family members or worried about “pestering” their relatives and friends (C14). In addition, 1 participant spoke about the forums being an antidote to their social isolation but that they were less important when they were able to speak to their friends in person (C10). For these participants, the forums played a powerful role in their lives by allowing them to speak and connect with people how and when they needed it most.

For some participants, the opportunity to connect with others provided a context in which they could understand or reframe their own experiences and develop hope for the future (C01 and C10):

I remember there was one guy that used to say, it does get better. It gets a lot better and I know that this is an awful feeling now, but it does get better and that was really big. That was life-changing.

C10

Participants also described others offering new skills or perspectives that they could then put to use in their own recovery and contextualize their experiences (C09 and C11). This practical support is discussed in detail in a later section of the Results.

#### Similar Experience

A strong theme reported among the respondents was the ability to connect with others who shared similar experiences. All but 1 of the participants (who did not speak about the topic) spoke about the utility of the forums being tied to the feeling that others had the same experiences as themselves. The participants felt that there was a large group out there that they could connect with, which made them feel less alone and allowed them to share with others who would understand their experiences—something that was rare in their everyday lives (C03, C04, and C07). This can be seen in the following quotation:

I wanted to be surrounded by people that know my experience rather than don’t know but want to help...Not therapists, not my best friends who has understanding but doesn’t have experience of it.

C04

This shared experience was a strong motivator for initial engagement with the forums. Ongoing engagement came from a sense of personal connections being made and a shared community around these experiences, which alleviated loneliness, developed a sense of belonging, and contributed a range of experiences and coping strategies. Moreover, 1 person went as far as to liken the connections they felt with people on the communities to the connections they had with their own family members (C12). Seeking out those with similar experience was also a means to normalize the experience of having a mental illness:

...it’s more the sense of other people relate to what you are saying and they can say yeah, I know exactly how you feel because this happened to me. You feel better about it all of a sudden.

C11

When I first went to hospital...I was feeling very isolated and pretty dysfunctional. Just like I was the only one and I just couldn’t cope or function as an adult. I went on there [the forums] and I had people say, you know what, we’ve been through this too and we’re better and it does get better.

C10

To find others with similar experiences, participants searched through forum threads to identify those that they most related to and which they could draw on for comfort, strategies, or a sense that someone understands (C01 and C08). Although many of these accounts spoke about receiving support from others, participants also spoke about the importance of providing support. This offered a way to use one’s experiences and knowledge to give back to others who supported them (C07, C08, and C12). It was also viewed as being “therapeutic” (C02) for the person providing the support.

For some, it was the connecting that was important rather than the content of what was discussed. The forums provided an opportunity to have a chat or socialize online with people who had something in common for people whose social isolation in the physical world meant that this type of day-to-day interaction was not otherwise possible. Participants spoke about forum events or get-togethers such as themed chat, “fun Fridays” (C15) or a “party” (C04) among online friends. In addition, 1 participant spoke about treating the forum as if they were chatting with friends or family over coffee:

...if you were living with somebody you would just say hi and here’s a coffee, whatever, so I feel like [the forum has] become that for me. If I sit down and have a coffee I’ll log on and that might be four times a day. Just the talking every day helps...chatting. Lifeline [a telephone based support service] isn’t always terribly helpful and they’re always in a rush.

C03

For this participant, this type of human, informal, interaction was not available from formal mental health services, which are more oriented to outcome and pressured for time. Another participant contrasted these types of interactions with in-person support groups, who they described in negative terms:

I used to go to pity parties, which are better known as support groups. They sit there and compare each other’s scars...I actually prefer the computer forum.

C09

Such interactions on the forums contributed to the connectedness of participants in their wider, offline communities (C04 and C10):

It’s been a big help in motivating me when you see people post tomorrow I’m going to go down and have a coffee. What are you doing tomorrow...I think I’ve learnt to speak better to people and that of course creates better relationships.

C04

Being able to actually articulate those experiences within that [online] community makes me more able to go out and talk about it to the broader community...It was a lot easier with something like bipolar to articulate it to the forums first before going out and talking to other people that really don’t understand it.

C10

As these quotations show participants spoke not only of an increased community connectedness but also a strength in communicating about the experience of mental illness and greater compassion for others in the community who may also have mental illness.

#### Boundaries, Anonymity, and Authenticity

Although the forums offered an opportunity for meaningful social connection, participants also recognized the need for boundaries. More than half of the participants mentioned that reading about other people’s circumstances could, on occasions, be difficult and could potentially “trigger” (C03 and C04) their own mental ill-health.

This meant that participants might need to establish and negotiate boundaries around how they used the forums, including allowing others to respond to certain posts rather than responding themselves or even staying off the forums, especially if they were feeling unwell:

I do self-regulate, because if it’s a similar situation sometimes it can be really beneficial and other times you’re just not in the headspace to hear someone else’s story the same.

C01

Participants also negotiated boundaries around what they were either prepared to discuss or were able to discuss due to restrictions on the posting of certain types of content by the site moderators. Moreover, 1 carer participant (C17) mentioned setting boundaries around what he would post because he was unsure if people should hear it.

Another participant described her inability to share suicidal ideation with someone in her community and then reflected that she could also not share this information with people on the forums. She reflected that having to negotiate the boundaries on what could be shared had the potential to be “tiring” and restricted her ability to be “open”:

You have to really think about how you share it. It gets a bit tiring when you’re not well.

C03

In addition to negotiating boundaries around difficult content, the requirement for anonymity imposed another boundary on participants. One participant called the requirement for anonymity a “double-edged sword” because it allowed them to “let a lot more out” but they had “to be a lot more careful about who knows who you are” (C08).

A sense of connection and community was both enabled and constrained by the need for anonymity. This was closely linked to the concept of authenticity. For some participants, the sense of being part of a genuinely supportive community was still authentic, despite anonymity. Moreover, 1 participant indicated that although she did not know people’s real names, she still felt like they were her friends (C04). Participants described the anonymity of the forums as “useful” (C10) and facilitating somewhere they could be more “real” (C03) and described impersonal nature of online social connection helpful for sharing personal information (C06).

Although anonymity made some feel they could be more real, it conversely made others feel that the connection to others was less real and the online anonymous format limited the extent to which they experienced a sense of connection and community (C02, C08, C14, and C17). This can be seen in the following quotations:

I don’t find it overly helpful to post there or talk there because it is remote. So something I’ve been looking for recently is a group in my town that we can go and even if we just go and have a cup of coffee and you’ve got people in front of you to talk to.

C02

I’ve never really counted people I’ve met on the internet as people in my life...That’s not [the forum’s] fault. That’s just how I think about the world.

C08

For these people, the forums played an important role but did not supplant their need for offline social support.

### Practical Advice and Information

Participants were directly asked about the importance of practical advice and information received via the forums. In addition to seeking social connection from people with similar experiences, participants stated that they accessed the forums to seek or provide practical information and advice (C01, C02, C03, C04, C08, C10, C11, and C14). C14 notes:

I would rather, because of the face to face factor, take the advice of [my] case manager...But, as I say, real life people and real life set ups like that, aren’t always available. In those circumstances in particular, [the] Forum can be excellent.

C14

The forum helped individuals with answering specific questions, developing a greater understanding of mental illness and by providing peer support.

#### Receiving Help for Specific Questions

The forums were used to ask or provide advice on specific questions. These questions were often practical in nature and related to how to navigate the mental health system, private health care (C01), or how to navigate other bureaucratic systems (C02). People also asked advice about other practical issues, which were impacted because of their mental ill-health, such as finding employment or housing:

At one stage I was investigating the concept of moving to Supported Residential Service Accommodation. I asked people on the forum if they had any experience...I got interesting feedback and useful feedback from that. The long and the short of it was that I didn’t end up moving.

C14

#### Greater Understanding of Mental Ill-Health Beyond Their Own Experiences

Participants mentioned that the forums had given them a greater understanding of mental illness, symptom management, and about where to get help (C03, C04, C08, C10, and C11). This came from reading other people’s posts, information and links posted by moderators, and information or discussion sessions linked to the forums:

I’ve got my two brothers are unwell. Just listening to how [people with] schizophrenia talk about their experiences. It just helps me understand my brothers a bit.

C03

I think it’s given me better insight into the diversity of experiences that can come under one diagnosis.

C10

I read people’s experiences across the board. I also look at links people have posted. That can be really educational. That’s made me do broader research on the internet, reading articles about mental health and all that kind of thing.

C11

This knowledge was often not directly relevant to their own personal experience but developed a valuable wider understanding of mental illness (C04 and C08). This breadth of knowledge helped with understanding others’ experiences and could be used to put one’s own experience into perspective.

However, others indicated that they already knew a great deal about mental illness and about the types of help available (C02). For these participants, the responses to their posts were supportive but not of much practical use. For some individuals, who had sought advice about specific issues, this could be disappointing, but for others, social connection was more important than advice, and involvement in the forums fulfilled other support roles in their lives (C11, C13, and C15):

What’s useful is mainly just knowing people are out there and they are compassionate...who can relate to what you are going through, that is supportive within itself...I don’t know if I really need advice as such.

C11

#### Professional Versus Peer Advice

In discussing the quality of advice and information provided through the forums, participants set up a distinction between peer advice and the advice provided by moderators. The forum moderators were viewed as a very important part of the forum experience by providing oversight and more “professional” support (C04, C07, and C08). One participant felt reassured that she could call on the moderators when she felt that another forum user might need more professional advice or a user had overdisclosed:

I love the fact that when I have concerns about a person...I can instantly send an email message and one of the [moderators] will then handle that. I can just say so and so, I’m concerned about their post, have a look...We know that there is someone professional out there that will get straight on and [remove their] phone number and their real name.

C04

However, some participants found the involvement of moderators or the posting guidelines too restrictive, which they felt limited the utility of the forums (C02 and C05), for example:

You can’t talk about this or that or treatment or anything like that. So there is quite a restrictive - I find it constricted but at least it’s an avenue for people to communicate to each other.

C05

Another participant reflected on the limits of boundary setting on the forums, which they felt had not gone far enough after a bad experience where moderators did not step in when needed (C07).

For another participant, the professional advice offered by one of the moderators was one of the highlights of the forum (C08). However, for this participant, it was important that the forum retains its function as a community of peers rather than as a site on which people could access advice from professionals:

I think if you have too many people doing it you’ll just turn people away a little bit, it’s meant to be a community for like-minded people. So you don’t want psychologist after psychologist in there.

C08

Several participants were concerned that the advice offered by peers was incorrect and felt that people with mental illness were not the best people to be giving advice on how to live well (C03, C05, C07, and C09).

Moreover, 1 participant mentioned that she did not like it when people “play doctor” (C09) and another was concerned that advice might be confusing because it was offered by “everyday people” with mental illnesses rather than professionals (C07). One participant (C05) wished to connect with individuals on the forum with a similar diagnosis but was concerned about the ability of someone who was unwell to offer support:

Because we are really isolated sometimes, and I guess that’s when we’re unwell and probably [when] we shouldn’t be communicating to another unwell person [laughs]. I don’t know.

C05

This shows that despite the use of the forums for social support, a number of the participants demonstrated a mistrust of lived experience and expressed stereotypical views about the ability of people who are mentally unwell to provide advice.

## Discussion

### Main Findings and Their Implications

This paper has developed our understanding of the experiences of engagement with general mental health forums in several key areas: (1) forum use by people living in rural and regional areas, (2) the value of peer support and peer education, (3) the experiences of carers in forums, and (4) the use of moderators. Each of these findings is discussed here in turn.

The study offers crucial insights into rural and remote mental health consumers and their motivations and experiences in using online forums. This study is based in Australia—a country which is highly urbanized but includes a large minority of people living in very sparsely populated regions where distances of hundreds of kilometers often separate people from support services for mental health. The interviews underscore that many participants experience considerable *social isolation and stigmatization* associated with their experiences of mental ill-health. This sense of isolation was especially acute for those participants in nonmetropolitan locations. The isolation compounded the pain, marginalization, exclusion, and other challenges that these participants experienced in relation to their mental ill-health. This isolation also cut participants off from the crucial social connection they felt that they needed and, which research has shown, will promote recovery [39].

The arguments for forums addressing social isolation arising from stigma have been well rehearsed elsewhere [17,20,25,40] but in only a very limited way in relation to people in rural and regional areas, for whom the issues of isolation and stigmatization are often more acute [21,41,42]. Although Australia has, in general, an adequate standard of mental health service provision through universal health care, in rural areas, services and supports are provided over long distances to small numbers of people, which means that all but the most essential medical services rarely operate outside of urban areas or major regional centers. These interviews show that it is not just a lack of appropriate services that leads to social isolation but both stigma and the “close-knit” context of small rural communities that stop people from connecting and receiving face-to-face support. An anonymous forum made sense to these participants because it was not place-based, and they were free to speak without people knowing who they were. The strong sense expressed by participants of sharing recognizably similar experiences with other participants in the forums was key to this sense of connection. Our findings support the findings of a relatively small, preliminary study of forum users in Australia, which found that online connections were able to address the isolation felt by people with mental ill-health in rural and regional areas [41]. More focused research on the experience of rural and remote forum participants is needed to confirm the findings presented here, but these results do provide preliminary support for forums as part of a national framework for rural and remote health.

The overwhelming message that emerged from the data was that for all the groups that accessed them—consumers and carers and urban and rural participants—the functioning of the forums as a site of connectedness was based on the value of peer support. There were 3 key components of this connection. As discussed in the previous sections, participants experienced considerable *social isolation*, which the forums helped to address as a way to find a *social connection* that was lacking in their everyday lives. Participants utilized the online social connections to both find and provide *support*, *information*, *and practical advice.* These are fundamental processes that have been identified strongly elsewhere in research on online forums more generally [24,35,43]. Our research adds to this by identifying the mechanisms by which forum involvement can enhance an understanding of the principals of recovery in the context of mental illness and enhance offline social inclusion.

Peer support is a core part of the recovery journey for many people experiencing mental ill-health [44]. This study revealed that peer support provided through the forums had 3 components: (1) individuals reflecting on the modeling of recovery or hope given by others sharing their stories (support of other to self); (2) the provision of support to others (support of self to other); and (3) visualizing individuals providing help to others on the forums (support of other to other). Several participants stated that the online community of the forums helped them to be more involved with their own communities in the offline world. For these participants, the “practice” of online discussion allowed them to develop a more powerful discourse of themselves and their mental ill-health, which they could use to counter perceived discourses of stigma that had hitherto stopped them from speaking. A similar finding has been noted previously in a study on forum use in postnatal mental ill-health, which found that the “virtual ‘voice’” which participants were able to express online “empowered them to disclose offline” [27]. This shows that the peer support provided by these forums may promote social inclusion in new and unexpected ways and, as such, should continue to be promoted as a key element of recovery-oriented mental health policy.

Previous research has largely ignored the inclusion of carers or family members in online support forums for mental health, except with some notable studies in the area of suicide bereavement support forums [45] and parenting children with mental ill-health [14,23]. Our results offer insights into why carers of people with mental illness engage with online forums, why they persist in doing so, and what benefits and issues they report that it brings them. When analyzing our results, we found that carer experiences largely mirrored those of participants in the mental illness “lived-experience”–focused forums and that half of the carers utilized both forums at the same time as they had a dual identity of both person with a lived experience of mental illness and carer. Although issues of stigma were noted less, the issues related to social isolation and need for connectedness were equally felt. Issues related to forum use more generally, including anonymity and boundary setting, were also equally felt among carer and lived experience forum users. These results demonstrate the necessity of including carer experiences in forum design. Large, general forums such as the one studied here, which focus on all types of diagnoses, as opposed to diagnosis-specific forums, makes it more likely that a large enough cohort of carers will be able to gather for a meaningful online community to develop.

The nature of the way information is circulated and advice is proffered raises issues for participants, especially in terms of accuracy, relevance, and appropriateness. Here, the availability, deployment, and intervention of experts—for instance, in the form of the moderators, specific advice from trained professionals, and opportunities for information sessions (such as webinars)—were often mentioned as important. The interviews show that such authoritative and trusted experts play a crucial role in the architecture, success, and sustainability of the forums, precisely because they provide a well-supported, welcome way to leaven and balance peer discussion with professional advice. Several international studies have also pointed to moderation as an important factor in maintaining a positive culture on forums [18]. Research that focuses on forums without moderation have emphasized the need for moderation to assist with participants who overdisclosed, broke forum posting rules, or were unkind to other participants [27,46]. However, content analysis of posts has also shown that participants were sometimes unhappy about moderator influence on their posts [20]. This demonstrates a need for moderation but in a form that is carefully crafted so that participants perceive it as a facilitator of support rather than a burden.

The participants expressed a range of views on anonymity and how it helped or detracted from their experience in the use of and attitudes toward the forums. For the majority, anonymity allowed them to express themselves in ways they were not able to do elsewhere—to be more “real” or “authentic.” This perception aligns with previous systematic reviews that have focused on the benefits of anonymous online self-disclosure [25,46]. For some, however, the anonymity, especially with an online platform, meant that they felt that the experience was less “real.” Previous research has also highlighted these 2 faces of anonymity [27,47]. Moreover, one antidote to this is to see the forums as one important element within a broader set of physical, virtual, and mixed environments of support [15]. Although it may be tempting to move all services online for this isolated community, online forums are not a panacea and services must complement rather than replace face-to-face services.

Another concern commonly raised by participants was the need to manage boundaries. Given the nature of online peer support, the burden falls upon consumers to manage themselves in various ways to make the best of the space while minimizing the potential negative implications of sharing their experiences. Several participants spoke of how they needed to ensure they were able to cope with what can be distressing communication or information from others, especially at times when they felt particularly vulnerable. It may be appropriate for forums to provide increased guidance for participants on this difficult balancing act, perhaps in the form of collaborative advice provided by other forum users and staff. “Peer knowledge” was not accepted uncritically by all. Although not a common theme, the comments that criticized the advice provided by others on the forums destabilized the idea of a peer support forum by restigmatizing people with mental illness as potentially inappropriate sources of information, advice, or social support more generally. Self-stigma has been noted in several other studies of online forums [21,48].

However, overall participation in the SANE forums was an important way for participants to learn about mental ill-health and, in doing so, challenge self-stigma. In addition, 2 other studies have also noted this benefit to participation [27,47]. Commentary that does engage in self or other stigmatizing can be addressed through explicit advice in forum rules or “pinned” posts, which explain stigma and provide advice on how it can be handled [21].

### Limitations

The findings of this study must be considered in relation to several limitations. These results relate to 2 specific forums, and therefore, participant responses relate to the structure and internal culture of those forums. Readers should peruse the forums to understand the context in which the study was written. Those that we spoke to were all registered users of the forums, as they had indicated their willingness to participate via a survey only sent to forum members. It is likely that those people who received the survey invitation were those that were motivated to become members rather than just read occasionally. Interview content may, therefore, be positively biased toward forum use and does not represent the views of those who only “lurk” or “listen” to the forum conversations.

### Conclusions

This research has offered important insights into the support- and information-seeking preferences and habits of mental health consumers and carers. It shows that online peer support provides a critical, ongoing role in providing social connection for mental health consumers. The online forums also provided crucial information and advice for many participants in this study, especially those living in rural and remote areas, who could not access this in their day-to-day lives.

There are important directions for future research that emerge from this qualitative study. Further work is needed to explore and understand the experiences of mental health forum participants living in rural and remote settings and to understand how online forums can be designed, promoted, maintained, and moderated to complement other available support, information, and interventions available offline or in other online formats.

## Appendix

Multimedia Appendix 1 Semi-structured interview schedule. Approved by ethics committee.
