# Supplementary material for: Social Connection and Online Engagement: Insights From Interviews With Users of a Mental Health Online Forum
Source: JMIR Ment Health. 2019 Mar 26;6(3):e11084. doi: 10.2196/11084 (PMC6454344; doi:10.2196/11084)
Supplement: Multimedia Appendix 1 [file mental_v6i3e11084_app1.pdf]

INTERVIEW QUESTIONS

Read to participant:

Thanks very much for agreeing to this interview.
My name is [NAME] and I am a trained researcher and mental health worker.
Before we start there are a couple of things that I need to check.
Do you have with you the Participant Information Sheet that we sent to you? Do you have any questions about the research after reading it?
Have you received a copy of the Participant Consent form. Could you please sign that form and email it back to me?

As the interview progresses I will ask you some questions about your mental health which may make you feel upset. If you feel distressed please let me know so that we can talk about what support you should access. You are free to stop the interview if you feel distressed at any time and you are free to refuse to answer any questions that upset you.
I am a trained mental health worker but I am also a member of the research team, which means that I can't offer you counselling but I can put you in touch with appropriate services should you need them.

Do you have someone that you can call for support if you feel distressed as a result of the interview? If not please note the support services listed in the participant information sheet and access these if you feel distressed.

Do you have any further questions before we start the interview?

Questions:

This is a semi-structured interview so while the questions will all follow the themes listed here the wording of the questions is only indicative of what will be asked.

1. When did you first access the SANE forums?
2. Why did you first decide to access the SANE forums?
3. Do you mainly access the lived experience forum or the carers forum?
4. How often do you use them?
5. What is the main issue that you are seeking support for?
6. How do you use them? (Prompt: for example do you just read the forums? Do you start posts? Do you answer other people's posts?)
   Prompts for further discussion depending on answer to 6:
   a) How do you feel about the other participants and their posts?
   b) What are the reasons that you respond to other people's posts?
   c) When others respond to your posts, do they offer you useful support or advice?
7. Has involvement in the SANE forums helped or hindered your mental health? Prompt: in what ways?
8. Do you think that the SANE forums have helped you to understand mental health more generally?
9. Do you think that you understand more about where to get help and what help is available for your mental health?
10. Do you feel more connected to the community as a result of your involvement in the SANE forums? (prompt: offline, online)
11. What do you like about the SANE forums?
12. What don't you like about the SANE forums?
13. Is there anything that you would like to see offered that is not currently offered through the SANE forums?
14. How do you think that SANE could better promote their forums?

15. Are you aware of any radio, television, social media promotions of the SANE Forums? If yes prompt: Do you think that promotion is effective?
16. How could they be made more effective?
